# Food-Derived Antidiabetic Peptides as Multi-Target Systemic Regulators: A Comprehensive Review of Sources, Preparation, Mechanisms and Future Perspectives

**DOI:** 10.3390/foods15122086

**Published:** 2026-06-09

**Authors:** Yiwei Yang, Ziwei Niu, Xiaohu Luo, Kang Chen, Xin Zhang, Lingling Jia

**Affiliations:** College of Food Science and Engineering, Ningbo University, Ningbo 315211, China; yyw2023032023@163.com (Y.Y.); m15839859807@163.com (Z.N.); luoxiaohu@nbu.edu.cn (X.L.); chenkang@nbu.edu.cn (K.C.); zhangxin@nbu.edu.cn (X.Z.)

**Keywords:** food-derived bioactive peptides, type 2 diabetes, DPP-IV inhibition, gut–pancreas axis, short-chain fatty acids, multi-target network regulation

## Abstract

Food-derived bioactive peptides have become a research hotspot in diabetes nutritional intervention due to their high safety, wide availability, and multi-target activities. This review addresses this by proposing a systems biology integration framework that defines these peptides as pleiotropic regulators of the gut microbiota-immune inflammation-metabolic signaling network, offering a novel systems-level perspective beyond previous reviews focused on single enzymes or pathways. The framework consists of three synergistic tiers. Tier 1 inhibits α-amylase, α-glucosidase or dipeptidyl peptidase-IV (DPP-IV) to control postprandial blood glucose. Tier 2 corrects insulin resistance by modulating phosphatidylinositol 3-kinase/protein kinase B (PI3K/Akt), activating nuclear factor erythroid 2-related factor 2 (Nrf2), and suppressing nuclear factor kappa-B (NF-κB). Tier 3 uses the gut as a hub to remotely coordinate metabolism via the gut–liver and gut–pancreas axes. The review also systematically summarizes the major sources and preparation methods of food-derived antidiabetic peptides, analyzes their advantages including multi-target network regulation, safety, and sustainability, as well as challenges such as oral bioavailability, insufficient clinical evidence, processing stability, and regulatory hurdles. Finally, it outlines future directions focusing on three actionable priorities: AI-assisted design, oral delivery systems, and high-quality clinical studies. This framework offers a new perspective for applying food-derived peptides in precision nutrition intervention for diabetes.

## 1. Introduction

Diabetes has become one of the top ten global diseases [1,2], with an estimated 853 million diabetic patients worldwide by 2050 [3,4]. The onset and progression of diabetes not only severely reduce patients’ quality of life but also impose a heavy economic burden on families and society [5,6]. The International Diabetes Federation (IDF) estimates that global healthcare expenditure related to diabetes reaches 760 billion USD annually, and it is predicted that these direct costs will increase to 825 billion USD by 2030 and to 845 billion USD by 2045 [7,8]. Therefore, safe, effective, and cost-effective dietary or nutraceutical approaches derived from natural food resources have emerged as a research hotspot for diabetes prevention and control.

**Figure 1:**
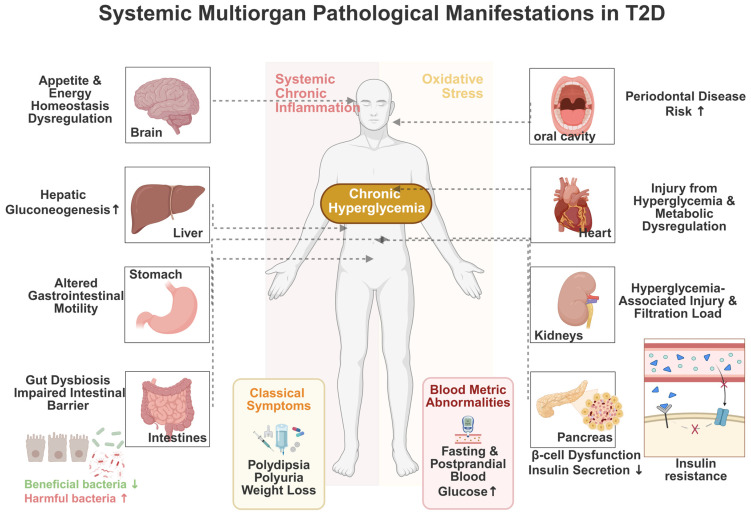
Systemic Multiorgan Pathological Manifestations in T2D. (Created with BioRender.com).

According to its pathogenesis, diabetes is mainly classified into type 1 diabetes (T1D) and type 2 diabetes (T2D). In addition, gestational diabetes mellitus (GDM) can occur under special conditions such as pregnancy [9,10]. T2D is the most common type, accounting for approximately 90% of all diabetic patients (Figure 1) [11]. It is primarily caused by insulin resistance, which reduces the body’s sensitivity to insulin [12], with its prevalence increasing across all age groups [13]. The onset of diabetes is co-regulated by multiple factors, including family genetics, environmental factors, and unhealthy lifestyles [14,15]. Numerous antidiabetic drugs are available, such as insulin degludec and metformin, and they play an important role in the management of diabetes, with therapy options constantly expanding. However, their long-term use could possibly lead to adverse reactions, including hypoglycemia, liver and kidney damage, and allergic reactions [16,17]. Given the escalating global prevalence of diabetes and the safety concerns associated with long-term pharmacotherapy, there is a pressing need for safer, multi-target adjuvant strategies derived from natural food resources. This rationale directly motivates the exploration of food-derived bioactive peptides as pleiotropic regulators of glucose metabolism [18,19].

Building on this rationale, food-derived bioactive peptides, specific amino acid sequences encoded in dietary proteins, have emerged as promising candidates. They are released from parent proteins by enzymatic hydrolysis, fermentation, or other methods, and can exert specific biological effects [20,21]. Numerous studies have confirmed that food-derived bioactive peptides possess various physiological activities such as antihypertensive, antioxidant, and immunomodulatory effects [22]. In recent years, the antidiabetic efficacy of food-derived bioactive peptides has gradually become a research focus [23]. In addition to traditional hotspots such as milk and legumes, antidiabetic potential has also been found in fruits, vegetables, plant seeds, marine fish, and other animal and plant sources [24]. A large body of evidence has demonstrated that these peptide segments act not only by directly inhibiting key hypoglycemic enzymes (e.g., α-amylase inhibition by salmon bone-derived peptide PIE [25] and α-glucosidase inhibition by mulberry leaf-derived peptide AAGRLPGY [26]), but also by systematically improving glucose homeostasis through multi-tiered mechanisms. These mechanisms encompass the modulation of insulin-related signaling pathways (e.g., activation of the PI3K/Akt/FOXO1 axis by milk-derived peptide LGP9 [27]), alleviating oxidative stress and inflammation (e.g., Nrf2 pathway activation by Bombay duck bone collagen peptide [28] and reduction in TNF-α levels by camel whey peptide [29]), and remodeling the gut microbiota (e.g., enrichment of butyrate-producing *Ruminococcus* by ginseng peptide) [30,31,32]. Representing the most extensively studied source categories (cereals, dairy, and marine products), these peptides collectively exemplify all three tiers of our proposed framework: direct enzyme inhibition (Tier 1), insulin signaling modulation (Tier 2), and gut microbiota remodeling (Tier 3).

A three-tier model of enzyme inhibition, insulin signaling, and gut axis has been applied to other natural products such as polyphenols and fiber. However, its application to peptides confers unique advantages. Peptides possess unique properties including sequence specificity, controlled enzymatic release, and the ability to serve as precursors for gut microbial metabolism. These properties elevate the three-tier model from a merely descriptive framework to a predictive and design-oriented tool for active intervention. Researchers can rationally design sequence-specific molecules targeting particular tiers, predict their multi-target effects, and optimize synergistic actions among different peptides. This paradigm shift from passive discovery to active design represents the central novelty of applying the three-tier model to food-derived antidiabetic peptides. Accordingly, the article first systematically summarizes the sources and preparation methods of food-derived antidiabetic peptides, then integrates their multi-target mechanisms of action including enzyme inhibition, signaling pathway modulation, and gut–organ axis regulation while elucidating the cross-talk among these mechanisms, and finally analyzes the advantages, challenges, and future research directions of these peptides.

### Search Methodology

To ensure a systematic and comprehensive synthesis of the current evidence, a structured literature search was conducted on food-derived bioactive peptides as systemic regulators of diabetes. The search was performed using the Web of Science, Scopus, and PubMed databases for relevant articles published up to February 2026. A predefined combination of keywords and Boolean operators was employed: (‘food-derived peptide’ OR ‘bioactive peptide’) AND (‘antidiabetic’ OR ‘hypoglycemic’ OR ‘DPP-IV inhibitor’ OR ‘α-glucosidase inhibition’ OR ‘insulin resistance’) AND (‘gut microbiota’ OR ‘gut-liver axis’ OR ‘gut-pancreas axis’ OR ‘short-chain fatty acids’).

The inclusion criteria comprised peer-reviewed original research articles and review papers published in English that investigated the sources, preparation, mechanisms, or efficacy of food-derived peptides in modulating diabetes-related pathways. Publications such as conference abstracts, patents, theses, and commentaries without original experimental data were excluded from the synthesis. Following a two-step screening process (title/abstract followed by full-text) conducted independently by two authors, a final set of literature was selected to construct the three-tiered network model and support the critical discussion presented in this review.

## 2. Sources and Preparation of Food-Derived Antidiabetic Peptides

### 2.1. Major Food Sources

The sources of antidiabetic food-derived peptides are extremely broad and can be roughly divided into plant sources, animal sources, and other emerging resources.

Peptides derived from plant sources such as cereals, legumes, and seeds are among the richest sources of inhibitory peptides against key digestive enzymes including α-glucosidase and dipeptidyl peptidase-IV (DPP-IV) [33,34], forming an important material reserve for the first tier (rapid front-end regulation) in postprandial glucose intervention. For example, X-Pro-type oligopeptides generated from wheat gluten hydrolysis are efficient DPP-IV inhibitors [35,36]; protein hydrolysates from millet, quinoa, and chia seeds have been reported to exhibit significant α-glucosidase or DPP-IV inhibitory activity [37,38,39,40]; and spent grain, a brewing by-product, is also a high-quality source of such peptides due to its high protein content [41]. Moreover, some plant peptides show potential for deep metabolic regulation (second tier). For instance, the pea peptide Vglycin upregulates insulin signaling pathway proteins [42,43], while the mung bean peptide has been shown to activate the AMP-activated protein kinase (AMPK) and phosphatidylinositol 3-kinase/protein kinase B (PI3K/Akt) pathways, suggesting dual rapid and long-term regulatory functions [44].

Peptides derived from animal sources, particularly dairy and meat proteins, are especially prominent in regulating insulin signaling pathways and alleviating systemic inflammation, thus participating more in the “deep intervention” (second tier) of diabetes pathology [45,46]. The bovine milk-derived peptide LGP9 is a classic example of regulating pancreatic β-cell function [47]. Goat milk, camel milk, and their fermented products (e.g., various cheeses) are rich in multiple DPP-IV and amylase inhibitory peptides, whose activity often increases with maturity, reflecting the impact of processing conditions [48,49,50,51]. DPP-IV inhibitory peptides can be released from beef, pork (especially collagen), and dry-cured ham, while egg white hydrolysate-derived peptides improve insulin resistance, often with anti-inflammatory properties [52,53,54].

**Figure 2:**
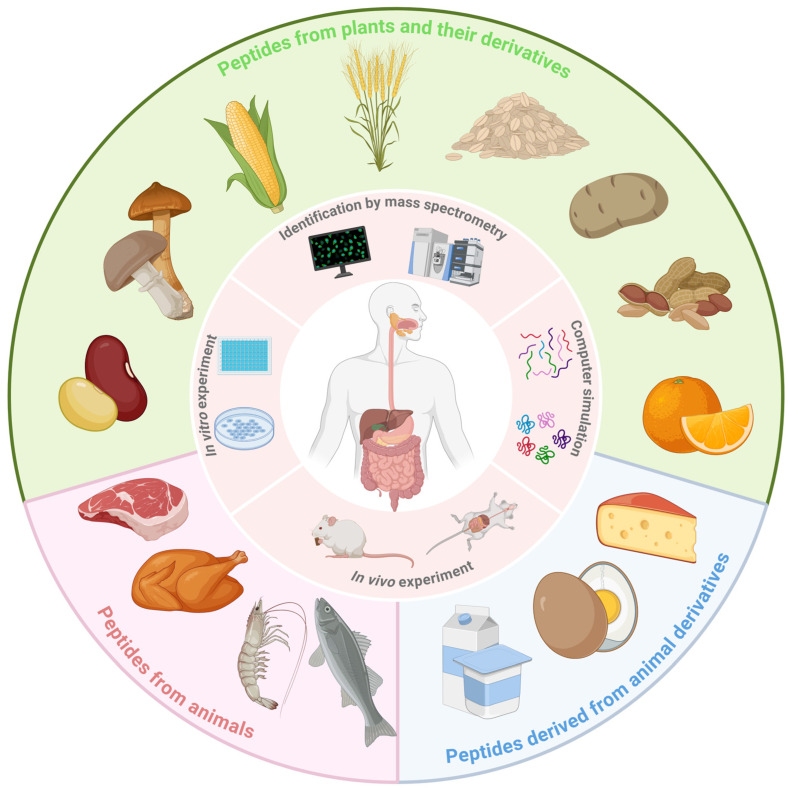
Sources and Identification Methods of Food-Derived Antidiabetic Peptides (Created with BioRender.com).

Marine and freshwater fish and their processing by-products are rich in Xaa-Pro sequences (which are easily recognized and bound by DPP-IV) within their collagen, making them a treasure trove of DPP-IV inhibitory peptides [55,56]. Hydrolysates from tilapia, skipjack tuna (PPP tripeptide), Baltic herring, and sardines are rich in potent DPP-IV inhibitory peptides [57,58,59]. Among them, the salmon bone-derived peptide PIE also exhibits excellent α-amylase inhibitory activity, demonstrating the multi-target potential (spanning Tiers 1 and 2) of aquatic peptides [25]. Numerous novel DPP-IV inhibitory peptides and insulin-releasing peptides have also been identified from gilthead seabream protein hydrolysates (Figure 2) [60].

### 2.2. Key Preparation Methods

The preparation of food-derived antidiabetic peptides is a critical step for releasing, enriching, or rationally designing specific functional sequences from natural proteins. Current main strategies include enzymatic hydrolysis, microbial fermentation, computer-aided rational design, and traditional food processing. These methods have different emphases and, together, constitute a toolbox for converting raw materials into active molecules [18].

Enzymatic hydrolysis is the most direct and controllable core technology. By selecting specific proteases (e.g., trypsin, alcalase, flavourzyme), proteins can be cleaved under mild conditions to release target peptides. Its advantage lies in high controllability: by optimizing enzyme type, temperature, pH, and hydrolysis time, a peptide profile rich in specific activities (e.g., DPP-IV inhibition or antioxidant activity) can be generated [61,62]. For example, ginger protease specifically hydrolyzes wheat gluten to release DPP-IV inhibitory peptides, while neutral protease treatment of silver carp protein yields peptide segments that resist gastrointestinal digestion and have better stability [63,64]. This method is suitable for large-scale preparation of well-defined “rapid front-end” inhibitory peptides [18]. However, achieving consistent outcomes based on this tunability is challenging in practice and for cross-study comparisons. Slight variations in critical parameters, such as the enzyme source, definition of activity units, and hydrolysis time, can lead to significantly different final peptide profiles and bioactivities, making direct comparison of results across different studies difficult. Nevertheless, the choice of protease directly determines the peptide profile and yield. For instance, trypsin, with its specificity for cleavage after arginine and lysine residues, tends to generate peptides with positively charged C-termini. Such peptides may exhibit stronger interactions with the negatively charged active pockets of enzymes like DPP-IV, thereby enhancing inhibitory specificity [65]. In contrast, alkaline proteases (e.g., alcalase) have broader cleavage specificity, often resulting in a higher degree of hydrolysis and peptide yield, but with a more complex product composition [66]. Flavourzyme can further hydrolyze bitter peptides, improving sensory properties alongside yield [67]. Consequently, for the preparation of peptides targeting specific bioactivities (e.g., potent DPP-IV inhibition), the rational selection of the optimal protease requires balancing yield and functional specificity, based on the amino acid sequence of the parent protein.

Microbial fermentation is a more complex synergistic system. Lactic acid bacteria, yeasts, or specific bacilli secrete abundant protease systems during fermentation; they not only hydrolyze proteins but also modify peptides (e.g., acetylation, glycosylation) through their metabolites, often producing structurally unique peptides with novel bioactivities [61,68,69]. For instance, fermentation of camel milk with *Citrobacter* and *Saccharomyces cerevisiae* produces multifunctional peptides with both antidiabetic and angiotensin-converting enzyme inhibitory activities [70,71]. Peptides prepared by fermentation have a natural affinity with gut microbial metabolites and are therefore ideal material sources for studying their regulation of the “gut-organ axis” [72]. However, the reproducibility of microbial fermentation is often constrained by key technical variables, such as genetic drift of the microbial strains, the use of complex or undefined media, and difficulties in precisely controlling fermentation parameters (e.g., minor fluctuations in dissolved oxygen, pH). These factors can lead to batch-to-batch variations in microbial metabolic profiles and proteolytic activities, thereby affecting the consistency of the final peptide profiles. To enhance reproducibility and controllability, future research could consider employing chemically defined media, standardized single or defined starter cultures, and integrating process analytical technology for real-time monitoring and feedback control of critical parameters.

Computer-aided rational design is emerging as a pioneering tool to break through traditional screening paradigms. Using molecular docking, machine learning, and quantitative structure-activity relationship (QSAR) models, large-scale virtual screening of food protein databases can be performed prior to experiments to predict and optimize potential peptide sequences with high affinity for targets such as DPP-IV and α-glucosidase [61,73,74]. For example, novel DPP-IV inhibitory peptides have been successfully predicted from beef, pork, and goat milk proteins [75,76]. However, although this method offers high prediction throughput, the success rate of translating virtual screening to experimental validation remains low. It is well established that only a small fraction of in silico hits are confirmed active in vitro, with even fewer proving efficacious in vivo. A comprehensive analysis of over 400 virtual screening studies reported a median hit rate of 13%, underscoring the generally low success rate of translating computational predictions into experimentally validated hits [77]. This critical limitation is rarely quantified in the literature, though the actual success rates are widely acknowledged to be very low. This disparity stems primarily from the limitations of current computational models: insufficient quality and scale of training data, inadequate simulation of peptide conformational dynamics and solvation effects, and the common neglect of critical pharmacokinetic parameters (e.g., absorption, distribution, metabolism, and excretion) [78]. Consequently, computational prediction primarily serves as a pre-screening tool to reduce experimental costs, and all its outcomes necessitate rigorous experimental validation [18]. The maturation and reliable application of this method ultimately depend on deeper interdisciplinary integration of bioinformatics, computational chemistry, and experimental biology.

It is worth noting that conventional food processing and cooking themselves constitute a natural “preparation” process. Heat treatment, endogenous enzyme action, and the use of starter cultures can all promote the release of active peptides from food proteins. For example, cooked millet shows better inhibitory activity after enzymatic hydrolysis, while the content and composition of DPP-IV inhibitory peptides in dry-cured meat products change dynamically during long-term ripening [52]. This observation suggests that optimization of daily dietary patterns itself may be a neglected strategy for active peptide intake [34]. However, this approach is inherently uncontrolled and cannot be standardized. Inter-individual differences in cooking methods, gastric digestion, and peptide release make it an unreliable delivery route. No data are available on how meal preparation variables affect peptide release or bioavailability. Therefore, this observation should be viewed as a hypothesis-generating insight rather than a validated strategy for active peptide intake.

After preparation, peptide sequences are typically identified using liquid chromatography-tandem mass spectrometry (LC-MS/MS) followed by database searching. For previously unreported peptides, de novo sequencing is usually applied [79]. Molecular docking and molecular dynamics simulations can be used to predict binding modes with target enzymes such as DPP-IV and α-glucosidase [80]. Validation can also be performed through in vitro enzymatic activity assays and, where feasible, in vivo efficacy studies. From mass spectrometry identification to computational prediction and experimental validation, these characterization methods are essential for establishing reliable structure-activity relationships.

In summary, the different preparation methods have their own pros and cons: enzymatic hydrolysis offers strong controllability but is often considered more expensive, though no quantitative cost comparison is available; fermentation can generate novel structures but suffers from poor reproducibility; computer simulation is high-throughput but has low validation rates; traditional processing is natural but uncontrollable. In the future, multiple strategies should be combined according to the functional requirements of the target peptides [61]. Without economic evaluations, industrial feasibility remains unsubstantiated.

## 3. Mechanisms of Antidiabetic Action of Food-Derived Peptides

**Figure 3:**
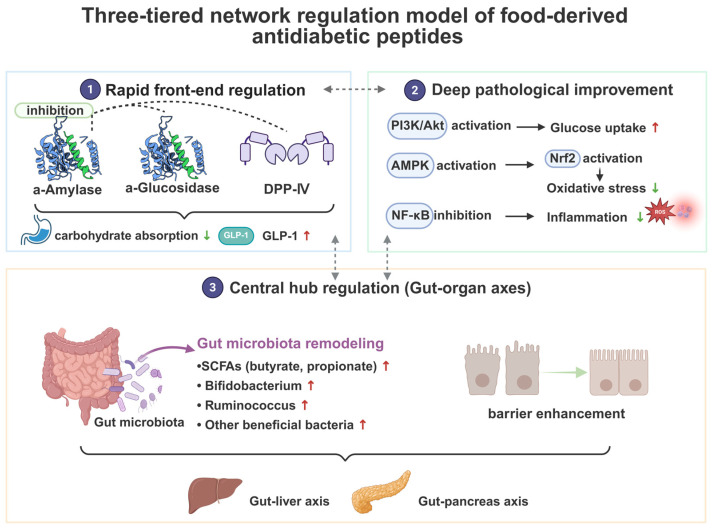
Three-tiered network regulation model of food-derived antidiabetic peptides (Created with BioRender.com). In the figure, red upward-pointing arrows (↑) denote an increase, rise, or enhancement (e.g., of microbial metabolites, beneficial bacteria, or glucose uptake). Green downward-pointing arrows (↓) indicate a decrease, reduction, or alleviation (e.g., of carbohydrate absorption, oxidative stress, or inflammation).

The antidiabetic efficacy of food-derived peptides stems not from potent inhibition of a single molecule but from their intrinsic capacity for multi-target, network-based systemic regulation. To coherently explain this, we integrate the available evidence into a three-tier physiological network model (Figure 3) [46,81]. Tier 1, termed rapid front-end regulation, provides immediate control of postprandial blood glucose by directly inhibiting α-amylase, α-glucosidase or DPP-IV [46]. Tier 2, deep pathological improvement, corrects the underlying metabolic soil of insulin resistance by modulating intracellular signaling pathways such as insulin/PI3K/Akt and alleviating oxidative stress and chronic inflammation [82,83].

Tier 3, central hub regulation, uses the gut as a hub to remotely coordinate the metabolism and immune status of the liver, pancreas, and other organs via gut microbiota remodeling, barrier function enhancement, and short-chain fatty acids (SCFAs) as signaling molecules through the gut–liver axis and gut–pancreas axis [84,85]. These three tiers are not linearly sequential but constitute a dynamic, interactive network with extensive cross-talk (Figure 3) [85,86].

### 3.1. Direct Regulation of Blood Glucose Through Inhibition of Digestive Enzymes and DPP-IV

Within the complex antidiabetic action network of food-derived peptides, direct inhibition of key metabolic enzymes constitutes the most defined and rapid defense line [23]. These peptides bind with high affinity to and inhibit key enzymes involved in either carbohydrate digestion or incretin degradation, thereby directly reducing postprandial blood glucose peaks [86] (Table 1). It should be noted that the IC_50_ values reported in Table 1 were measured under different assay conditions across studies; the values are presented solely to illustrate the range of reported activities. Moreover, for most peptides listed, the inhibition mechanism (competitive, non-competitive, or mixed) remains uncharacterized. Future studies should include kinetic analyses to define inhibition modes and guide peptide design.

Food-derived peptides delay carbohydrate absorption primarily by inhibiting the key digestive enzymes α-amylase and α-glucosidase [23]. For example, the salmon bone-derived peptide IEELEEELEAER (PIE) has an IC_50_ of approximately 100 μg/mL, achieving an inhibition rate of 58.81% at this concentration. In-depth mechanistic studies showed that this peptide stably binds to the enzyme’s active center through hydrophobic interactions, inducing conformational rearrangement that significantly reduces the α-helix content and increases β-sheet content, thereby directly disrupting catalytic function [25]. Similarly, the peptides AAGRLPGY and RWPFFAFM identified from mulberry leaf protein have been confirmed to inhibit α-glucosidase with IC_50_ values in the micromolar range, exhibiting in vitro inhibitory potential [26]. This action mimics the pharmacological principle of classical carbohydrate blockers, but because the peptides are derived from food proteins, they are considered to have superior safety for long-term consumption.

Regarding the regulation of the incretin system, food-derived peptides efficiently target DPP-IV. This enzyme degrades glucagon-like peptide-1 (GLP-1), thereby shortening its insulin-releasing activity [46]. Numerous studies have identified potent DPP-IV inhibitory peptides from diverse food sources. For instance, the peptides LTWR and DPF isolated from *Musculus senhousei* were confirmed as competitive inhibitors with inhibition constants in the low micromolar range [116]. Molecular docking further revealed interaction details: goat milk-derived peptides YPF and LLLP form dense hydrogen bond and hydrophobic interaction networks with key residues (e.g., ARG358, PHE357) in the DPP-IV active pocket, efficiently blocking substrate entry [117]. Notably, fish gelatin is an excellent precursor of such inhibitory peptides because its collagen is rich in Xaa-Pro sequences, and proline residues represent the ideal structure for both DPP-IV cleavage preference and inhibition [118,119]. These in silico and in vitro findings are valuable for identifying candidate peptides. For example, oral administration of a specific peptide fraction from Atlantic salmon skin gelatin significantly reduced plasma DPP-IV activity, increased active GLP-1 levels by approximately 1.5-fold, and improved oral glucose tolerance in diabetic model rats [107]. However, it should be noted that most in vivo studies used pre-hydrolyzed peptide mixtures rather than single pure peptides, which may mask activity differences among individual peptides. A more critical issue is that the use of complex mixtures makes it difficult to attribute observed biological effects to specific peptide sequences. This lack of sequence-level resolution obscures structure-activity relationships and limits the ability to predict which structural features confer activity. Furthermore, potential synergistic or antagonistic interactions among co-existing peptides are rarely examined. Progress in the field will therefore require dose–response comparisons between purified single peptides and their parent hydrolysates, as well as systematic structure-activity studies to clarify the contribution of individual sequences.

From a physiological perspective, native GLP-1 has a plasma half-life of only 1–2 min due to rapid degradation by DPP-IV [120]. Even with complete DPP-IV inhibition, the increase in active GLP-1 is ultimately limited by its secretion rate. Clinical DPP-IV inhibitors (e.g., sitagliptin) achieve >80% DPP-IV inhibition and elevate meal-stimulated active GLP-1 levels by 2- to 3-fold [121]. In contrast, most food-derived DPP-IV inhibitory peptides exhibit modest inhibition (typically 20–40% in vitro) and have unknown inhibitory potency in humans. Whether such modest inhibition can meaningfully raise active GLP-1 levels in humans remains unclear. Future studies should directly measure active GLP-1 concentrations after peptide consumption to clarify this. Nevertheless, a critical and more general issue is that potent in vitro DPP-IV inhibition does not always translate into in vivo efficacy. For example, a tilapia skin gelatin peptide with strong in vitro activity failed to lower blood glucose in rats due to poor absorption [63]. This does not invalidate in vitro screening, which remains an essential first step for identifying candidate peptides. However, for most peptides, in vivo data are lacking, and the correlation between in vitro activity and glucose-lowering effect remains unestablished. Future studies should therefore complement in vitro assays with pharmacokinetic and in vivo efficacy evaluations.

Collectively, although the sequences of identified inhibitory peptides vary greatly, their highly active members are often rich in hydrophobic amino acids (e.g., Pro, Leu, Phe) or positively charged residues, which facilitate binding to the enzyme active pocket. However, systematic quantitative structure-activity relationship (QSAR) studies in this field are still lacking. Moreover, the in vivo validation rate for peptides predicted by computational methods remains low [122,123].

### 3.2. Improvement of Insulin Resistance Through Signaling Pathway and Oxidative Stress Regulation

The core mechanism by which food-derived peptides improve insulin resistance lies in their multi-level systemic intervention in upstream causes-namely, insulin signaling pathway dysregulation, oxidative stress, and chronic inflammation. This “deep intervention” (Tier 2) aims to restore tissue insulin sensitivity and normal metabolic function at the root [46,124] (Table 2).

At the insulin signaling pathway level, several food-derived peptides precisely target and correct the cascade from receptor to downstream kinases [46]. The milk-derived peptide LGP9 directly reverses pancreatic β-cell dedifferentiation in diabetic models and promotes islet structure and function repair by activating the PI3K/Akt/FOXO1 signaling axis, demonstrating a direct protective effect on endocrine organs [27]. For improving insulin sensitivity in peripheral tissues, the pea peptide Vglycin and the marine collagen peptide GPAGPHGPPGKDGR enhance glucose uptake efficiency in target tissues such as skeletal muscle by upregulating insulin receptor phosphorylation or activating the PI3K/Akt/mTORC2 axis, respectively [42,129]. Notably, some peptides (e.g., the pea peptide LLPHF) are designed to bypass the already resistant insulin receptor and directly activate downstream Akt, providing a new approach for severe insulin-resistant states [130]. Peptide sequences that mimic or enhance insulin signaling often contain clusters of basic amino acids or hydrophobic cores, which may mediate interactions with cell membranes or signaling proteins [131,132,133]. However, this research area faces a fundamental challenge: most evidence comes from cell experiments using high peptide concentrations that are typically much higher than those achievable in vivo [134]. It is also important to recognize that the peptide concentrations routinely used in cell-based assays (often in the range of 100–1000 μg/mL) are substantially higher than the plasma levels that can be realistically achieved after oral administration, which typically remain in the low μg/mL or even ng/mL range [135]. As highlighted by Foltz et al., a valid research approach should demonstrate peptide stability toward peptidases and evaluate its ADME properties, and future in vitro studies should use physiologically relevant concentrations and times [136]. Therefore, future work should aim to use concentrations closer to physiological exposure and, whenever feasible, confirm critical observations in animal models with measured circulating peptide levels.

In terms of alleviating oxidative stress and chronic inflammation, food-derived peptides also work by activating endogenous defense systems and inhibiting key inflammatory pathways, thereby clearing the “metabolic soil” of insulin resistance [137,138]. For example, bone collagen peptide from Bombay duck (*Harpadon nehereus*) significantly activates Nrf2, a central regulator of cellular antioxidant defense, and upregulates the expression of its downstream target proteins HO-1 and NQO1, enhancing cellular antioxidant capacity from the source [28]. Meanwhile, camel whey protein peptides effectively reduce serum levels of key pro-inflammatory cytokines such as TNF-α and alleviate tissue inflammatory infiltration by promoting macrophage polarization toward an anti-inflammatory phenotype [29]. Many studies also report radical scavenging activity using cell-free assays such as DPPH and ABTS. These assays measure chemical antioxidant capacity and are useful for initial screening. However, they do not predict in vivo antioxidant activity due to poor oral bioavailability and rapid metabolism of peptides. Therefore, results from cell-free assays should be interpreted as chemical properties rather than evidence of physiological effects, and they must be distinguished from cell-based or in vivo evidence of Nrf2 activation or oxidative stress reduction. The antioxidant activity of such peptides is often associated with the presence of electron-donating amino acids (e.g., tyrosine, tryptophan) in their sequences, while their anti-inflammatory effects may involve direct interference with specific inflammatory signaling pathways [131,139].

A cautionary note is warranted regarding the clinical translation of antioxidant peptides. The history of antioxidant interventions in diabetes, such as vitamin E and vitamin C, has been largely disappointing, with most large-scale clinical trials failing to demonstrate consistent glycemic benefits despite promising in vitro and animal data [140]. It is not self-evident that peptide antioxidants will succeed where small molecules have failed. However, peptides may offer distinct advantages, such as sequence-specific activation of the Nrf2 pathway, modulation of gut microbiota, and potential synergistic effects with other bioactive components. These potential advantages, however, remain largely hypothetical and require rigorous clinical validation. Therefore, while food-derived antioxidant peptides hold promise, their clinical efficacy should not be assumed based on in vitro radical scavenging data alone.

Another underexplored aspect is tissue specificity. Insulin resistance manifests differently in liver, muscle, and adipose tissue, as extensively reviewed [141,142]. Yet most studies treat improved insulin sensitivity as a single entity without distinguishing which tissues respond. Furthermore, whether orally administered peptides distribute preferentially to specific tissues remains unknown. Future research should assess tissue-specific responses (e.g., glucose uptake in muscle versus suppression of gluconeogenesis in liver) and, where technically feasible, measure peptide distribution to identify primary sites of action [143].

A more fundamental methodological limitation persists even if effects are attributed to direct action: the validation of peptide engagement with specific signaling pathways (e.g., PI3K/Akt, Nrf2). The current state of research indicates that claims of pathway activation remain largely reliant on correlative evidence such as Western blot analysis of phosphorylated proteins, and have not sufficiently employed tools like isoform-specific inhibitors or CRISPR-knockout models to establish necessary causality and confirm the functional necessity of these targets. Consequently, observed changes in protein phosphorylation or nuclear translocation themselves constitute high-level correlative evidence, which could also stem from broader cellular stress or metabolic reprogramming. To translate the systemic regulatory advantages of food-derived peptides into predictable therapies, future research must employ more sophisticated experimental strategies to establish unambiguous causality. This requires not only disentangling direct from indirect effects but also utilizing genetic or pharmacological loss-of-function approaches to verify whether specific targets are indispensable for the peptide-mediated benefits.

In summary, the evidence presented in this section regarding insulin signaling, antioxidant, and anti-inflammatory pathways collectively outlines the potential of food-derived peptides to correct deep-seated pathologies. However, a crucial methodological issue is distinguishing whether the observed effects originate from direct peptide actions or are indirect consequences of overall metabolic improvement. For instance, a reduction in serum inflammatory factors (e.g., TNF-α, IL-6) could result from direct peptide inhibition of inflammatory pathways like NF-κB, or it could occur indirectly following the prior amelioration of hyperglycemia and insulin resistance, which alleviates inflammatory drivers. The correlative data (e.g., biomarker changes) provided by most current studies are insufficient to rigorously define this causality.

### 3.3. Regulation of the Gut–Organ Axis via Gut Microbiota and Barrier Function Modulation

The intervention of food-derived peptides in diabetes profoundly reflects their systemic, remote-regulatory properties with the gut as the central hub. By precisely remodeling the gut microbiota, enhancing intestinal barrier function, and modulating the resulting key signaling molecules, food-derived peptides coordinate the metabolic activities of the liver and pancreas, embodying the essence of their “multi-target network regulation” [23,81,86] (Table 3).

The action begins with functional ecological reconstruction of the gut microbiota. This regulation is not a simple increase or decrease in bacterial numbers but a selective enrichment of microbiota with specific beneficial metabolic functions [81,147]. For example, ginseng peptide intervention significantly increased the abundance of butyrate-producing bacteria such as *Ruminococcus* in the gut of T2D mice, while walnut protein-derived peptides enriched bacteria from the families *Lachnospiraceae* and *Ruminococcaceae* [148]. These bacteria are key producers of SCFAs, especially butyrate and propionate. Therefore, peptide regulation essentially enhances the gut’s capacity to synthesize beneficial metabolites, laying the material foundation for subsequent remote signaling via the gut–organ axes (Tier 3) [149].

Thus, by selectively enriching functional bacteria such as butyrate producers, food-derived peptides directly elevate the levels of SCFAs like butyrate in the gut. This shift in metabolite concentration is the critical step that translates changes in microbial composition into physiological effects. Nevertheless, several points deserve caution. Butyrate elevations reported in the cited studies are modest and were measured in feces rather than portal blood. Whether such increases can reach concentrations sufficient for systemic effects, such as histone deacetylase inhibition (typically requiring mM levels), is unclear, as no direct measurements of portal butyrate have been performed. Therefore, based on the available data, the physiological relevance of these changes remains uncertain. Future studies should measure portal or circulating SCFA levels to better assess their systemic impact and, where feasible, employ stable isotope tracing to assess butyrate production and flux.

A healthy microbiota is inseparable from intact intestinal barrier function. Dysbiosis disrupts intestinal mucosal integrity, leading to endotoxin translocation that drives systemic chronic inflammation and insulin resistance. Food-derived peptides help consolidate the barrier primarily through the microbiota remodeling described above [147,148,149]. Butyrate, as the preferred energy source for colonocytes, directly promotes the expression and assembly of tight junction proteins (e.g., Occludin, ZO-1). Thus, by enriching butyrate-producing bacteria, peptides indirectly strengthen the intestinal barrier. At the same time, the anti-inflammatory properties of the peptides themselves help alleviate local intestinal inflammation, creating a favorable environment for barrier repair [150]. A stable intestinal barrier reduces metabolic endotoxemia at its source and alleviates systemic low-grade inflammation [151].

The intestinal signaling molecules produced by these processes, particularly SCFAs, serve as core messengers for remote dialogue via the gut–liver axis and gut–pancreas axis [81,149]. In the gut–liver axis, butyrate and propionate are taken up by the liver via the portal circulation. Butyrate acts as a histone deacetylase inhibitor, regulating hepatic lipid metabolism genes through epigenetic mechanisms; propionate activates the hepatic AMPK signaling pathway, directly inhibiting gluconeogenesis [151]. However, HDAC inhibition by butyrate typically requires millimolar (mM) concentrations, whereas systemic butyrate levels in portal blood are usually in the micromolar (μM) range [152]. Therefore, it is unlikely that butyrate exerts significant HDAC inhibition systemically. Instead, butyrate may act locally at high concentrations in the colonic epithelium or, more plausibly under systemic conditions, via G-protein coupled receptors (e.g., GPR41, GPR43), which are activated at much lower concentrations [153]. Future studies should clarify which mechanism predominates under physiological conditions. Studies have shown that milk-derived glycomacropeptide precisely improves hepatic insulin sensitivity by enriching butyrate-producing bacteria and elevating host butyrate levels [145,146]. Additionally, some peptides modulate microbiota-related bile acid metabolism, affecting secondary bile acid levels and thereby regulating the glucose and lipid metabolic network through nuclear receptors such as the farnesoid X receptor in the liver [154]. In the gut–pancreas axis, butyrate is one of the most potent inducers of GLP-1 secretion from intestinal L cells. Consequently, food-derived peptides that enrich butyrate-producing bacteria essentially enhance GLP-1 synthesis and release at the source. This mechanism forms a potential upstream-downstream synergy with classical DPP-IV inhibitory peptides (e.g., skipjack tuna-derived PPP tripeptide) that inhibit GLP-1 degradation in the circulation. The increased GLP-1 promotes insulin secretion in a glucose-dependent manner and protects β-cell function, thus precisely reinforcing the gut–pancreas dialogue [86]. However, it is important to note that GLP-1 secretion is not solely regulated by microbiota-derived SCFAs; vagal nerves and nutrient sensors (e.g., GPRC6A, CasR) also play dominant roles [155,156]. The present review focuses on the microbiota-SCFA axis, but a complete picture of gut–pancreas communication requires integration of neural and other signaling pathways. Future studies should consider these additional regulatory layers.

Nevertheless, this frontier area still faces challenges in deep mechanistic dissection. Most current studies focus on observing overall changes in microbiota composition and metabolites, but little is known about the specific molecular processes of peptide-microbiota interactions (e.g., how peptides are recognized and utilized by specific bacterial species). Moreover, research often remains at the level of microbiota structural description, with insufficient attention to the regulation of their functional genome and metabolic pathways, limiting precise interpretation of how the microbiota affects host metabolism [157].

A critical limitation is that most cited gut microbiota studies are correlative. They demonstrate changes in bacterial abundance (e.g., *Ruminococcus*, *Lachnospiraceae*) following peptide treatment, but no study has used germ-free mice or fecal microbiota transplantation (FMT) to prove that these microbial shifts cause the observed metabolic benefits. Causality therefore remains unestablished. Future studies should incorporate FMT from peptide-treated donors into germ-free recipients or use defined microbial consortia to directly test whether microbiota changes are sufficient to improve glucose homeostasis.

An additional critical gap is that for peptides to reach and modulate gut microbiota, they must survive transit through the stomach and small intestine. However, data on peptide stability in simulated gastric fluid (SGF) and intestinal fluid (SIF) are lacking for nearly all sequences discussed in this review. Although some studies have investigated peptide stability in gastrointestinal (GI) fluid [158], such data are not available for the antidiabetic peptides reviewed here. Direct evidence of peptide-microbe interactions remains scarce [159]. Without such data, claims of direct microbiota modulation remain speculative. Future studies should include in vitro digestion stability assays and, where possible, co-culture experiments of peptides with relevant gut bacteria to assess direct peptide-microbe interactions.

### 3.4. Cross-Talk and Synergistic Effects Among the Three Tiers

The three tiers described above are not isolated or linearly progressive but constitute a dynamic, interactive network mediated by various molecular signals (Table 4) [84]. First, “rapid front-end” DPP-IV inhibitory peptides (e.g., PPP) not only inhibit GLP-1 degradation but some have also been found to directly improve insulin signaling, exemplifying multi-target functionality [160,161]. Second, anti-inflammatory and antioxidant peptides, while improving peripheral insulin sensitivity, also help maintain intestinal barrier integrity, reduce endotoxin translocation, and thereby alleviate systemic inflammation. This establishes a positive feedback loop between Tier 2 (deep intervention) and Tier 3 (gut hub) [162,163]. Third, butyrate produced by the gut microbiota not only stimulates GLP-1 secretion from L cells but also regulates hepatic gluconeogenic gene expression through epigenetic mechanisms, thereby simultaneously amplifying the incretin effect (Tier 1) and improving hepatic insulin sensitivity (Tier 2) [161,164]. This cross-level synergy may enable low doses of a single peptide or combinations of multiple peptides to produce a sum effect greater than that of a single mechanism [160,165]. However, such synergistic interactions remain largely qualitative claims; few studies have quantitatively evaluated their contributions, and predictive tools based on network pharmacology or systems biology models are currently lacking [166,167].

Although current research is predominantly derived from in vitro and animal models, several human clinical trials underscore the translational potential of food-derived antidiabetic peptides. For example, whey protein trials primarily used postprandial glucose and insulin as primary outcomes [168]; marine collagen peptide studies focused on fasting glucose and HbA1c change [169]; and a study on salmon skin gelatin hydrolysate reported increased active GLP-1 levels (approximately 1.5-fold) as a mechanistic endpoint [107]. Soybean peptides, milk protein hydrolysates, and marine collagen peptides have significantly improved postprandial blood glucose, insulin sensitivity, and GLP-1 levels in prediabetic and T2D patients [168,170,171,172]. However, the clinical evidence base remains limited. Nevertheless, large-scale, long-term, randomized double-blind controlled trials (RCTs) are still scarce, and dose–response relationships and long-term safety require further validation [170,173]. Moreover, the described cross-talk and potential synergy among the three regulatory tiers are currently entirely qualitative. No synergy index (e.g., Chou-Talalay combination index), network pharmacology model, or dose–response matrix has been reported for any peptide combination. It therefore remains unknown whether the combined effect of multiple tiers genuinely exceeds the sum of individual effects. Future research should adopt quantitative approaches, including median-effect analysis and systems modeling, to evaluate synergy and guide rational peptide combinations. A more fundamental limitation is the heavy reliance on cell lines and rodent models, which differ from human physiology in gut microbiota composition, enzyme expression, and immune regulation. Positive results in animals do not guarantee human efficacy. To bridge this translational gap, studies should prioritize human validation and, where possible, use human tissue or organoid models to better predict clinical responses.

## 4. Advantages and Challenges

### 4.1. Advantages

Based on an integrated analysis of existing studies, food-derived antidiabetic peptides exhibit multiple core advantages that distinguish them from traditional chemical drugs [124]. Their most fundamental value lies in their multi-target, network-based mode of action, enabling them to simultaneously intervene in multiple key nodes of the diabetic pathological network: directly and rapidly inhibiting digestive enzymes to control postprandial blood glucose, deeply modulating insulin signaling pathways and alleviating oxidative stress and chronic inflammation to correct the root causes of insulin resistance, and remodeling the gut microbiota to achieve systemic remote regulation via the gut–organ axis [46]. This systemic intervention potential against a complex metabolic disease may generate synergistic effects and reduce the compensatory risks associated with potent inhibition of a single pathway [174].

Another major advantage is the favorable safety profile of food-derived peptides, as they originate from natural food proteins. Their preliminary safety has been validated in multiple preclinical and early-stage clinical studies [175]. For example, the lactoferrin-derived peptide Lactoferricin B has shown very low acute toxicity and good tolerability in animal experiments [176]. Certain marine collagen peptides at doses far exceeding the effective level did not exhibit obvious organ toxicity or abnormal blood biochemical parameters [177]. Moreover, specific antidiabetic peptides derived from common allergens such as rice and soybean did not elicit unexpected allergic reactions in particular studies [178]. These systematic safety assessments provide experimental support for their low risk upon long-term consumption and lay the foundation for their high acceptability as functional foods or dietary supplements integrated into daily diets [18]. Nevertheless, potential allergic reactions deserve attention, as many sources (camel milk, egg white, wheat gluten, peanut, cricket) are common allergens. Diabetic patients who consume these peptides daily over long periods may face sensitization risks. Therefore, allergenicity assessments (e.g., IgE binding, digestive stability) should be included in future studies.

Furthermore, the raw material sources for these peptides are extremely broad and sustainable. Not only can traditional animal and plant protein resources be utilized, but food processing by-products (e.g., fish skin, bones, okara) can also be efficiently exploited, thereby converting potential waste into high-value ingredients and conferring both economic and environmental advantages for large-scale production [174,179]. Collectively, these advantages constitute the unique competitiveness and broad application prospects of food-derived peptides as a novel nutritional intervention strategy for diabetes.

### 4.2. Challenges

Despite the promising prospects, the path of food-derived antidiabetic peptides from “potential molecules” to “clinical products” faces a series of severe scientific and engineering challenges [180].

Practical challenges related to raw material supply are often overlooked. Salmon bone, Bombay duck, turtle egg, and insect sources face obstacles including seasonal variability, high purification costs, and, for some species, conservation concerns. Economic analyses are lacking, and scalability remains uncertain. Future work should consider underutilized by-products as more sustainable alternatives. Natural sources vary with season, diet, and processing, yet standardized quality control for peptide extracts is lacking. Batch-to-batch variability hampers cross-study comparison and industrial reproducibility. Reference materials, standardized protocols, and routine quality control (e.g., chromatographic fingerprinting) are urgently needed [181].

A primary challenge is the fundamental limitation of oral bioavailability. Peptides are prone to degradation by gastrointestinal proteases and have low absorption efficiency across the intestinal barrier [182,183]. Consequently, many peptides that exhibit high activity in vitro fail to achieve the expected efficacy after oral administration [184]. Most food-derived peptides show very low oral bioavailability in preclinical models, and even therapeutically optimized peptides typically achieve only about 1% oral bioavailability [185]. Currently, pharmacokinetic data (Cmax, t_1/2_, AUC) remain unavailable for the vast majority of sequences reported in the literature. For example, Fleury et al. compared the DPP-IV inhibitory potential of seven food proteins both in vitro and in vivo in rat plasma after oral administration and found no correlation between in vitro and in vivo results [186]. This finding underscores that without pharmacokinetic data, in vitro activity alone cannot predict oral efficacy. Moreover, Abeer et al. pointed out that current in vitro permeability assays have limitations in predicting oral bioavailability in humans, and there are difficulties in relating the low blood levels detected in preclinical models to human-relevant pharmacodynamic readouts [187]. This absence of quantitative pharmacokinetic data means that, for most peptides, it is unknown how much intact peptide reaches systemic circulation or target tissues, making it difficult to determine effective doses. Therefore, mechanistic claims derived solely from in vitro or indirect in vivo studies remain speculative until such data are obtained. Proposed solutions such as nanoencapsulation and self-emulsifying drug delivery systems (SEDDS), although promising, still lack human validation for the specific peptides discussed here.

A further challenge is the relatively low potency of many food-derived peptides. As shown in Table 1, Table 2 and Table 3, the half-maximal inhibitory concentrations (IC_50_) of numerous reported active peptides are in the microgram per milliliter (μg/mL) to milligram per milliliter (mg/mL) range. For instance, the PIE peptide from salmon bone exhibits an IC_50_ against α-amylase of approximately 100 μg/mL, which is typically several orders of magnitude higher (i.e., less potent) in molar concentration than clinical drugs like acarbose (with an IC_50_ in the nanomolar range). This potency gap implies that achieving biochemical inhibition comparable to drugs at a single target might require impractically high peptide intake, posing significant challenges for oral bioavailability, formulation cost, and potential gastrointestinal tolerability. Therefore, the “multi-target” characteristic should not be viewed merely as a compensatory mechanism for “low potency” but rather as a distinct mode of action: by moderately and synergistically modulating a network of related targets, it may be possible to elicit a beneficial physiological reset with lower occupancy at any single site. Consequently, a key future direction is to enhance potency at critical targets through rational design, structural optimization, or innovative delivery systems, while preserving the multi-target profile.

An additional difficulty lies in translating fundamental discoveries into practical applications, as the current evidence chain suffers from insufficient depth and completeness. Most mechanistic studies rely on cell and rodent models, which differ from the complex pathology of human diabetes. Many peptides require long-term administration in animal experiments to observe modest improvements. However, whether such mild, pleiotropic effects are clearly effective in humans, the required doses, and long-term safety all lack high-quality human clinical trial evidence. At present, well-designed, adequately powered clinical studies are very limited [170,188].

Another significant obstacle is the practical constraint posed by food matrices and processing conditions when moving from laboratory research to industrialization. Peptides ultimately need to be delivered through foods or specific formulations. Food is a complex system where other components (e.g., fats, minerals) may interact with peptides, affecting their activity and stability [189]. Processing conditions such as heat treatment (>60 °C), pH changes, and high salt concentrations can also destroy peptide structure and lead to inactivation. For example, whey protein peptides tend to precipitate in acidic beverages, while collagen peptides can lose more than 40% of their activity after heat treatment (>70 °C, 30 min) [190]. There is currently a lack of systematic databases on processing stability [180]. Moreover, many protein hydrolysates or peptides themselves have undesirable flavors (e.g., bitterness) mainly due to terminal hydrophobic amino acids, which affects product sensory acceptability. Masking techniques (e.g., cyclodextrin encapsulation, enzymatic debittering, Maillard reaction modification) need to be developed [191,192]. A recent study systematically evaluated peptide stability under various thermal processing conditions [193], but comprehensive data for antidiabetic peptides are still lacking. Future research should prioritize the development of stability databases and predictive models to guide formulation and processing.

Another consideration is that multi-target action, while promoted as a benefit, may also lead to off-target or unintended on-target but unwanted effects. For example, excessive GLP-1 secretion, whether directly stimulated by peptides or indirectly via butyrate, could cause gastrointestinal adverse effects such as nausea, vomiting, and delayed gastric emptying [194]. Similarly, potent inhibition of α-glucosidase or α-amylase, the primary mechanism for postprandial glucose control-can result in carbohydrate malabsorption, leading to flatulence, abdominal bloating, and diarrhea. These symptoms are well documented for clinical α-glucosidase inhibitors (e.g., acarbose). Although food-derived peptides are generally considered safer, the possibility of similar side effects at higher doses or with chronic consumption cannot be excluded. Future studies should systematically monitor gastrointestinal tolerability and other potential adverse effects in long-term animal studies and clinical trials.

Importantly, all safety data are from acute or subacute studies (days to weeks). No long-term (6–12 months) carcinogenicity or chronic toxicity data exist for any of the peptides listed in this review. Consequently, the safety of their chronic daily consumption remains unknown and requires future investigation.

Finally, regulatory hurdles add to commercialization uncertainty. The regulatory classification of functional peptides differs among countries and regions. Before industrialization, it is necessary to clarify the positioning as a food, health food, or novel food ingredient to reduce compliance risks [61]. To provide clearer guidance, three common regulatory pathways are briefly distinguished. In the United States, a GRAS (Generally Recognized as Safe) notification confirms that a substance is safe for its intended use in food, based on scientific procedures or common use in food prior to 1958. This pathway is suitable for peptides intended as food ingredients or dietary supplements. In the European Union, a Novel Food application is required for foods or food ingredients that were not consumed significantly in the EU before May 1997; this involves a safety assessment by the European Food Safety Authority (EFSA). In China, a “new food ingredient” approval from the National Health Commission is required. By contrast, if a peptide is intended for therapeutic use (e.g., glycemic control in diabetic patients), it would need to be approved as a drug (e.g., FDA New Drug Application, NDA) [FDA] [195]. To date, no food-derived antidiabetic peptide has received regulatory endorsement specifically for diabetes management under any of these pathways. This uncertainty poses a major barrier to commercialization, and early consultation with regulatory agencies is advisable for product development.

## 5. Conclusions and Future Perspectives

### 5.1. Conclusions

This review demonstrates that the key strength of food-derived bioactive peptides is their multi-target, network-based systemic regulation. Through the synergistic modulation of digestive enzyme activity, insulin signaling pathways, oxidative stress, inflammatory status, and critical gut–organ axis crosstalk, they intervene at multiple levels within the complex pathological network of diabetes. This integrated mode of action positions them as a particularly promising strategy for nutritional intervention in diabetes management. However, translating these discoveries from the laboratory to clinical application necessitates critical advances in overcoming fundamental challenges, most notably in oral delivery, processing stability, and the generation of robust clinical evidence. Addressing these hurdles is paramount to unlocking the full multi-target network potential of food-derived peptides and transforming their theoretical promise into tangible clinical practice.

### 5.2. Future Perspectives

Although food-derived antidiabetic peptides exhibit unique advantages in multi-target network regulation, translating them from laboratory discovery to practical application still faces many challenges [180]. To fully realize their potential, future research should be strategically directed towards the following critical areas: (1) Rational design and high-throughput screening: Integrate artificial intelligence (e.g., deep learning, molecular docking) with multi-omics technologies to efficiently predict and optimize peptide sequences with desirable multi-target activities from food protein databases, and establish public bioactive peptide databases [73,196,197]. However, it must be acknowledged that current models are trained on small, biased datasets and have not yet yielded a clinically validated antidiabetic peptide. Therefore, prediction accuracy requires rigorous assessment, and all computational hits demand thorough experimental validation. (2) Innovative oral delivery systems: Develop novel technologies such as nanoencapsulation, enteric-coated microspheres, and SEDDS to enhance gastrointestinal stability, transmembrane absorption efficiency, and targeted delivery of peptides [183,198]. (3) High-quality human clinical evidence: Conduct multicenter, randomized, double-blind, placebo-controlled trials. For short-term trials (weeks to months), postprandial and fasting glucose should serve as primary endpoints; glycated hemoglobin (HbA1c) is best reserved as a key secondary endpoint for trials lasting ≥6 months [170,199]. (4) Considerations for industrialization: Investigate the impact of food processing (e.g., heat treatment, pH, ionic strength) on peptide activity, develop debittering and taste-masking technologies, and clarify regulatory pathways across different jurisdictions to facilitate product development and market entry [192,200,201].

Furthermore, a critical concentration disconnect exists in many in vitro cellular studies elucidating the mechanisms of peptide action. To observe significant effects, numerous experiments employ high peptide concentrations in the range of 100–1000 μg/mL in models such as HepG2 hepatocytes or L6 myotubes. These concentrations far exceed the levels reasonably achievable in human plasma following oral intake via diet or supplements (typically in the ng/mL to low μg/mL range). The pathway activation (e.g., PI3K/Akt) or anti-inflammatory effects observed at such “supraphysiological” concentrations may not occur at true physiological or pharmacological doses, potentially leading to an over-optimistic estimation of peptide potency and mechanisms of action. Future research should place greater emphasis on validating mechanisms within pharmacokinetically relevant concentration ranges and should couple such studies with investigations into the absorption, distribution, metabolism, and excretion of these peptides to define the in vivo fate of active constituents. This integrated approach is crucial for improving the predictability of findings from cellular models to whole-animal and human studies.

In addition, existing evidence in this field carries inherent limitations in statistical and methodological rigor, warranting cautious interpretation. First, a majority of preclinical studies (particularly animal experiments) employ small sample sizes (e.g., n = 5–8 per group) and generally lack a priori power calculations. This may result in underpowered studies that fail to detect true biological effects or inflate the role of chance in the observed outcomes. Second, many studies measure a large number of outcome variables and perform multiple statistical comparisons without appropriate correction for false discovery rate (e.g., using Bonferroni or FDR correction). This substantially increases the risk of reporting “statistically significant” findings by chance alone. Therefore, future high-quality research should adhere to principles of preregistration, sample size estimation, and correction for multiple comparisons to generate more reliable and reproducible conclusions, thereby providing a solid evidence base for the systemic effects of food-derived peptides. Through these efforts, food-derived antidiabetic peptides are expected to truly move from laboratory discovery to functional food or dietary supplement applications.

## Figures and Tables

**Table 1:** Food-Derived Peptides Targeting Tier 1: Rapid Front-End Regulation (Direct Enzyme Inhibition).

Source	Peptide/Component	Target/Pathway	Key Activity Data	In Vivo Data	Inhibition Type	Reference
Wheat gluten	X-Pro type oligopeptides	DPP-IV inhibition	Potent DPP-IV inhibitory activity; specifically released by ginger protease	-	Competitive (PPFS), Mixed (APFGL, PPFW)	[87]
Foxtail millet prolamin	Hydrolyzed peptide fraction	α-Glucosidase inhibition	Significant α-glucosidase inhibitory activity; superior effect after cooking	-	-	[88]
Amaranth seed	Peptides with >13 amino acids	DPP-IV inhibition	Binds to dimerization site of DPP-IV, blocking active dimer formation	-	-	[89]
Quinoa protein	Hydrolysate (MW < 1 kDa)	DPP-IV inhibition	Excellent DPP-IV inhibitory activity; can improve glucose intolerance in gestational diabetic mice	Gestational diabetic mice, oral, hydrolysate mixture	-	[64]
Pinto bean	Pro-Pro-His-Met-Leu-Pro, etc.	α-Amylase and DPP-IV inhibition	Exhibits dual inhibitory activity against α-amylase and DPP-IV	-	-	[90]
Mulberry leaf	AAGRLPGY, RWPFFAFM	α-Glucosidase inhibition	IC_50_ in the low millimolar range (0.6–1.2 mg/mL) for α-glucosidase	-	-	[26]
Rice wine lees	YPLPR	α-Glucosidase inhibition	IC_50_ 5.31 mmol/L; suggests excellent inhibitory activity	-	-	[91]
Olive seed	Hydrolyzed peptide fraction	DPP-IV inhibition and GLP-1 stability	Not only inhibits DPP-IV but also enhances GLP-1 secretion; acts on gut–pancreas axis	-	-	[92]
Goat milk	Hydrolyzed/fermented peptide fractions	α-Amylase inhibition and AMPK activation	Dozens of peptides exhibit α-amylase inhibition; also activates AMPK in liver and muscle	Streptozotocin-induced diabetic rats, oral, fermented/hydrolyzed mixtures	-	[93]
Camel milk	Hydrolyzed peptide fractions	DPP-IV and α-Amylase inhibition	Enzymatic treatment releases potent DPP-IV and α-amylase inhibitors	-	-	[94]
Fermented camel milk	Multiple peptides (e.g., SSHPYLEQLY)	α-Amylase and α-Glucosidase inhibition	Exhibits inhibitory activity against both enzymes; derived from microbial fermentation	-	-	[95]
Tulum cheese	β-casein, κ-casein derived peptides	DPP-IV inhibition	Contains DPP-IV inhibitory peptides; content increases with cheese ripening	-	-	[96]
Parmesan cheese	Peptide fractions from different ripening stages	α-Amylase, α-Glucosidase and DPP-IV inhibition	Exhibits multi-target inhibitory activities	-	-	[97]
Beef/Pork protein	Peptides identified via in silico analysis	DPP-IV inhibition	Novel DPP-IV inhibitory peptides predicted; collagen is a high-quality precursor	-	-	[98]
Frozen-then-aged beef	Peptides released after digestion	DPP-IV, α-Glucosidase and ACE inhibition	Highest digestibility and releases diverse bioactive peptides	-	-	[99]
Spanish dry-cured ham	Specific sequences (not indicated)	DPP-IV inhibition	DPP-IV inhibitory activity detected	-	-	[100]
Dry-cured pork tenderloin	Peptide fractions at different ripening days	DPP-IV inhibition	Most abundant at 90, 270 days and 1 year; activity enhanced by LAB inoculation	-	-	[101]
Chicken foot protein	p4H (peptide fraction)	DPP-IV inhibition and GLP-1 release	Stimulates GLP-1 release from intestinal endocrine cells	-	-	[102]
Turtle egg protein	Enzymatic oligopeptides	α-Glucosidase inhibition	Competitively inhibits α-glucosidase; stable upon simulated gastrointestinal digestion	-	Competitive	[103]
Mealworm (*Alphitobius diaperinus*)	Enzymatic hydrolysate	α-Glucosidase inhibition	Exhibits α-glucosidase inhibitory, antioxidant and antihypertensive activities	-	-	[104]
Yellow mealworm (*Tenebrio molitor*)	LPDQWDWR (among others)	DPP-IV inhibition	Flavourzyme hydrolysate shows excellent DPP-IV inhibitory activity	-	-	[105]
Cricket (*Gryllodes sigillatus*)	Alkaline protease hydrolysate	DPP-IV, α-Amylase and α-Glucosidase inhibition	Multi-target inhibitory activities; highlights potential of edible insect peptides	-	-	[106]
Tilapia skin gelatin	Ginger protease hydrolysates (e.g., GPXGPPGPGP)	DPP-IV inhibition	Strongest in silico DPP-IV inhibitory activity; poor GI stability and low oral absorption	Rat oral absorption study	-	[63]
Atlantic salmon skin gelatin	Peptide fraction for animal study	DPP-IV inhibition and GLP-1 release	5-week intervention lowered plasma DPP-IV activity, increased active GLP-1 ~1.5-fold	Diabetic model rats, oral, peptide fraction (mixture)	-	[107,108]
Atlantic salmon processing by-products	10 identified peptides	DPP-IV inhibition	Exhibits DPP-IV inhibitory activity	-	-	[109]
Tuna cooking juice	Specific sequences (not indicated)	DPP-IV inhibition	Derived from cooking processing by-products	-	-	[110]
Tuna skeletal myosin	LADW, EEAEGT	α-Glucosidase inhibition	Shows α-glucosidase inhibitory potential; certain GI digestion tolerance	-	-	[111]
Skipjack tuna muscle protein	PPP (tripeptide)	DPP-IV inhibition (also PI3K/Akt and AMPK activation)	Strongest DPP-IV inhibitory activity in vitro; also alleviates insulin resistance	IR-HepG2 cells only	-	[58]
Silver carp protein	Neutral protease hydrolysates (e.g., APGPAGP)	DPP-IV inhibition	Strongest DPP-IV inhibitory activity; stable upon simulated GI digestion	-	Competitive/Non-competitive mixed	[112,113]
Baltic herring and by-products	Enzymatic hydrolysates (e.g., BHSSH)	DPP-IV inhibition	Comparable to Atlantic salmon skin gelatin hydrolysates	-	-	[114]
Sardine by-product	Enzymatic peptides (e.g., NAPNPR)	DPP-IV inhibition	Derived from fishery processing by-products	-	-	[115]
Square bream (Capros aper) protein	22 novel DPP-IV inhibitory peptides	DPP-IV inhibition	In vitro DPP-IV inhibitory activity; stable to GI digestion; stimulates insulin secretion	-	-	[60]
Salmon bone	IEELEEELEAER (PIE)	α-Amylase inhibition	IC_50_ ≈ 100 μg/mL, inhibition rate 58.81%; binds to active site, induces conformational change; also inhibits adipocyte differentiation	-	-	[25]

**Table 2:** Food-Derived Peptides Targeting Tier 2: Deep Pathological Improvement.

Source	Peptide/Component	Target/Pathway	Key Activity Data	In Vivo Data	Reference
Buckwheat	AFYRW	Insulin signaling and glucose metabolism	Suppresses hepatocyte steatosis and triglycerides; ameliorates insulin resistance; alters pancreatic protein glycosylation	HFD/STZ-induced diabetic mice, oral, pure peptide	[125]
Pea	Vglycin	Upregulates insulin receptor (p-IR) and Akt (p-Akt)	Improves insulin sensitivity, with effects comparable to physiological doses of insulin; directly enhances glucose tolerance	STZ/HFD-induced diabetic rats, 80 mg/kg/day, 4 weeks, oral, pure peptide; also tested in HFD-fed C57BL/6J mice (15 weeks) and T2D C57BL/6 mice (8 weeks)	[43]
Mung bean	HTL (tripeptide)	AMPK signaling pathway	Promotes cellular glucose uptake via AMPK activation; promotes GLUT4 translocation	-	[44]
Mung bean	FLSSTEAQQSY, TLVNPDGRDSY	PI3K/Akt signaling pathway	Improves glucose metabolism; activates PI3K/Akt; ameliorates glucose metabolism disorders in diabetic models	HFD-induced insulin-resistant mice, 245 mg/kg/day, 5 weeks, dietary intervention, peptide mixture (not single peptide)	[44]
Peanut protein	TH fraction (contains specific sequences)	Anti-diabetic activity (pathway not detailed)	Contains peptide sequences with anti-diabetic activity; also exhibits free radical scavenging activity	-	[126]
Cow’s milk	LGP9	PI3K/Akt/FOXO1 signaling	Improves pancreatic β-cell dedifferentiation and promotes islet repair in T2D mice; direct protective effects on pancreatic islets	T2D mice (HFD/STZ-induced), 1, 3, and 9 mg/kg/day, 4 weeks, oral, pure peptide; T1D mice (alloxan-induced), 0.3 and 1 mg/kg, 4 weeks, pure peptide	[27]
Goat milk	Hydrolyzed/fermented peptide fractions	α-Amylase inhibition (T1) and AMPK activation	Activates AMPK in liver and skeletal muscle; improves systemic glucose homeostasis in diabetic rats	STZ-induced diabetic rats, oral, fermented/hydrolyzed mixtures	[93]
Egg white	QAMPFRVTEQE (Peptide 2)	Ameliorates insulin resistance (IR)	Improves HFD-induced obese and IR mice; effects superior to rosiglitazone; reduces hepatic lipid deposition	Diet-induced insulin resistant SD rats, oral, pure peptide	[127]
Amphibian skin secretions	Various antimicrobial/active peptides	Stimulates insulin secretion	Stimulates insulin release in vitro at low concentrations with low cytotoxicity; serve as design models for synthetic optimized peptides	-	[128]
Bombay duck bone	Collagen peptide mixture	Nrf2/HO-1 pathway	Upregulates HO-1 and NQO1 expression; enhances cellular antioxidant capacity	STZ-induced T1D mice, 240 mg/kg, oral, peptide mixture (HNCP, <1 kDa)	[28]
Camel whey	Hydrolysate	TNF-α, macrophage polarization	Reduces serum TNF-α, alleviates inflammation; promotes anti-inflammatory macrophage polarization	-	[29]
Skipjack tuna muscle	PPP (tripeptide)	DPP-IV inhibition (T1) and PI3K/Akt/AMPK	Alleviates insulin resistance in IR-HepG2 cells; activates both PI3K/Akt and AMPK pathways	IR-HepG2 cells only	[58]

**Table 3:** Food-Derived Peptides Targeting Tier 3: Central Hub Regulation via Gut–Organ Axes.

Source	Peptide/Component	Target/Pathway	Key Activity Data	In Vivo Data	Reference
Ginseng	Ginseng peptide (GP)	Gut microbiota (enrichment of butyrate-producing *Ruminococcus*)	Increases butyrate levels, improves glucose tolerance; reverses dysbiosis in T2D mice	db/db mice, 30 and 60 mg/kg, 6 weeks, oral gavage (tube-feeding), peptide fraction [mixture containing oligopeptides]	[32]
Walnut protein	LPF	Gut microbiota (enriches *Lachnospiraceae* and *Ruminococcaceae*)	Increases *Firmicutes*/*Bacteroidetes ratio*; reduces harmful *Bilophila*; modulates gut microbiota in colitic mice	DSS-induced colitis mice, 100 mg/kg, oral, pure peptide (LPF)	[144]
Bovine milk	Casein glycomacropeptide (CGMP)	Gut microbiota (butyrate producers) and hepatic insulin sensitivity	Enriches butyrate-producing bacteria, elevates host butyrate levels, improves hepatic insulin sensitivity	High-fat, high-fructose diet-fed mice, 12 weeks, dietary intervention, pure peptide (GMP); prediabetic human volunteers	[145,146]
Olive seed	Hydrolyzed peptide fraction	Inhibits DPP-IV and enhances GLP-1 stability/secretion (gut–pancreas axis)	Simultaneously inhibits DPP-IV (T1) and promotes GLP-1 secretion; multi-faceted regulation of incretin system	-	[92]
Chicken foot	p4H (peptide fraction)	Inhibits DPP-IV and stimulates GLP-1 release from intestinal L cells	Inhibits DPP-IV (T1) and directly stimulates GLP-1 secretion; acts on gut–pancreas axis	Diet- and age-induced glucose-intolerant rats, 300 mg/kg body weight, acute oral administration, protein hydrolysate (mixture)	[102]
Skipjack tuna	PPP (tripeptide)	Inhibits DPP-IV (elevates active GLP-1) and improves insulin signaling	Potent DPP-IV inhibitor (T1); also activates PI3K/Akt and AMPK pathways (T2); indirectly enhances GLP-1 action	IR-HepG2 cells only	[58]

**Table 4:** Summary of the three-tier network regulation model for food-derived antidiabetic peptides.

Tier	Primary Targets/Mechanisms	Representative Peptides	Key Effects	Cross-Talk Mediators
Tier 1	α-amylase, α-glucosidase, DPP-IV	PIE (salmon), AAGRLPGY (mulberry), PPP (tuna)	Reduced postprandial glucose	GLP-1, incretin effect
Tier 2	PI3K/Akt, AMPK, Nrf2, NF-κB	LGP9 (milk), Vglycin (pea), HTL (mung bean)	Improved insulin sensitivity, antioxidant, anti-inflammatory	SCFAs, reactive oxygen species
Tier 3	Gut microbiota (butyrate producers), gut barrier, gut–liver/pancreas axes	Ginseng peptide, walnut LPF, camel whey hydrolysate	Remote metabolic regulation via SCFAs, GLP-1	Butyrate, propionate, secondary bile acids

## Data Availability

No new data were created or analyzed in this study.

## Abbreviations

The following abbreviations are used in this manuscript:

DPP-IV	Dipeptidyl peptidase-IV
GLP-1	Glucagon-like peptide-1
PI3K	Phosphatidylinositol 3-kinase
Akt	Protein kinase B (PKB)
Nrf2	Nuclear factor erythroid 2-related factor 2
NF-κB	Nuclear factor kappa-B
SCFA	Short-chain fatty acid
IC_50_	Half-maximal inhibitory concentration
AMPK	AMP-activated protein kinase
GDM	Gestational diabetes mellitus
HO-1	Heme oxygenase-1
NQO1	NAD(P)H:quinone oxidoreductase 1
IDF	International Diabetes Federation
HFD	High-fat diet
STZ	Streptozotocin
HbA1c	Glycated hemoglobin
